# South Asian populations in Canada: migration and mental health

**DOI:** 10.1186/1471-244X-14-154

**Published:** 2014-05-26

**Authors:** Farah Islam, Nazilla Khanlou, Hala Tamim

**Affiliations:** 1 School of Kinesiology and Health Science, York University, 357 Bethune College mailroom, 4700 Keele Street, Toronto, ON M3J 1P3, Canada; 2 School of Nursing, York University, Toronto, ON M3J 1P3, Canada

**Keywords:** Mental health, Mental illness, South Asian, Canada, Immigrant, Canadian-born, First generation immigrant, Second generation immigrant, Migration, Social determinants of mental health

## Abstract

**Background:**

South Asian populations are the largest visible minority group in Canada; however, there is very little information on the mental health of these populations. The objective of this study was to determine the prevalence rates and characteristics of mental health outcomes for South Asian first-generation immigrant and second-generation Canadian-born populations.

**Methods:**

The Canadian Community Health Survey (CCHS) 2011 was used to calculate the estimated prevalence rates of the following mental health outcomes: mood disorders, anxiety disorders, fair-poor self-perceived mental health status, and extremely stressful life stress. The characteristics associated with these four mental health outcomes were determined through multivariate logistic regression analysis of merged CCHS 2007–2011 data.

**Results:**

South Asian Canadian-born (3.5%, 95% CI 3.4-3.6%) and South Asian immigrant populations (3.5%, 95% CI 3.5-3.5%) did not vary significantly in estimated prevalence rates of mood disorders. However, South Asian immigrants experienced higher estimated prevalence rates of diagnosed anxiety disorders (3.4%, 95% CI 3.4-3.5 vs. 1.1%, 95% CI 1.1-1.1%) and self-reported extremely stressful life stress (2.6%, 95% CI 2.6-2.7% vs. 2.4%, 95% CI 2.3-2.4%) compared to their Canadian-born counterparts. Lastly, South Asian Canadian-born populations had a higher estimated prevalence rate of poor-fair self-perceived mental health status (4.4%, 95% CI 4.3-4.5%) compared to their immigrant counterparts (3.4%, 95% CI 3.3-3.4%). Different profiles of mental health determinants emerged for South Asian Canadian-born and immigrant populations. Female gender, having no children under the age of 12 in the household, food insecurity, poor-fair self-rated health status, being a current smoker, immigrating to Canada before adulthood, and taking the CCHS survey in either English or French was associated with greater risk of negative mental health outcomes for South Asian immigrant populations, while not being currently employed, having a regular medical doctor, and inactive physical activity level were associated with greater risk for South Asian Canadian-born populations.

**Conclusions:**

Mental health outreach programs need to be cognizant of the differences in prevalence rates and characteristics of mental health outcomes for South Asian immigrant and Canadian-born populations to better tailor mental health services to be responsive to the unique mental health needs of South Asian populations in Canada.

## Background

### South Asian populations in Canada

This study’s objective was to determine the prevalence rates and characteristics associated with mental health outcomes for South Asian populations in Canada. A multiplicity of definitions of “South Asian” exists in Canada. Definitions are based on ancestral origins, culture, language, religion, and geopolitical boundaries, to name a few. Statistics Canada’s Canadian Community Health Survey (CCHS) defines South Asian as those who self-identify having ancestors who are “South Asian (e.g. East Indian, Pakistani, Sri Lankan)” [1]. According to the 2006 Census [2], over 1.26 million people from South Asian populations called Canada home (4.0% of the total population), making up the largest visible minority group in Canada [3]. The majority of those reporting South Asian origin in the 2001 Census were foreign-born (68%), while only about a third were Canadian-born (32%) [3]. The Census defines “South Asian” as those who self-identify as having “ancestry that originates in South Asia, including those reporting their origin as at least one of Bangladeshi, Bengali, East Indian, Goan, Gujarati, Kashmiri, Pakistani, Punjabi, Nepali, Sinhalese, Sri Lankan, Tamil, or South Asian” [4]. About three quarters of the foreign-born South Asian population were recent immigrants, arriving in Canada in the last 20 years [5]. In 2011, over a quarter of a million people immigrated to Canada as permanent residents [6]. About 15% emigrated from South Asia: 24,965 from India (10%), 6,073 from Pakistan (2%), 3,104 from Sri Lanka (1%), 2,449 from Bangladesh (1%), and 1,249 from Nepal (0.5%) [6]. It is projected that by 2031, 55% of Canada’s foreign-born population will report origins in Asia [7].

### Mental health of South Asian populations in Canada

One in five Canadians will experience a mental illness or addiction during their lifetime [8]. The economic cost of mental illnesses in Canada is staggering. It was estimated that the direct and indirect costs of mental illness was at least $51 billion in 2003 [9]. Mental health is a particular area of concern for South Asian populations in Canada. Health sector workers and members of South Asian communities in Toronto, Ontario identified mental health as a highly stigmatized and silenced health issue within South Asian populations [10]. South Asian individuals with major depressive episode reported the highest proportion (48%) of unmet mental healthcare need and highest proportion (33%) of perception of barriers to the availability of mental healthcare compared to eight other ethnic groupings in Canada [11]. Few studies have examined prevalence rates of depression in South Asian populations, and inconsistency exists within the literature. While analyses of the National Population Health Survey (NPHS) found lower rates of depressive symptoms and major depression (prevalence rates not reported) [12,13], a study of older adult South Asians in Calgary found more than double the prevalence rate of mild depression (21%) compared to the national average (10%) [14]. Hierarchical regression analysis revealed that being female, poorer self-perceived health, lower physical health, and a higher level of agreement with South Asian cultural values increased the likelihood of depression for older adult South Asians in Calgary, Alberta [15].

### Migration as a determinant of mental health

Health is determined by a broad set of factors beyond health care and lifestyle choices [15]. “Social” factors such as income, social status, gender, and social support networks also impact upon health [15]. Both premigration (circumstances leading to migration, i.e. in the cases of war trauma for refugees) and postmigration factors (loss of social status, social support, separation from family, difficulty integrating into a new culture, and lack of employment) can be sources of stress for newcomers (Beiser & Edwards, [16]). Migration and culture are important social determinants of mental health [16,17]. In an open-ended survey, where South Asian populations in Toronto were asked to write in their responses to a questionnaire, migration and the culture clash between the parental first generation of immigrants and second generation of South Asian youth were identified as risk factors for mental health and sources of stress, anxiety, depression, and identity loss [10]. Moreover, migration stress, low income and loss of social status, poor social networks, low education and literacy, unemployment and difficult working conditions, language barriers, and older age were all outlined as risk factors of mental health for immigrants (and additionally for refugee, ethnocultural, and racialized groups) [17].

Furthermore, there is evidence for a short-lived “healthy immigrant effect” for mental health, where immigrants converge to national-born levels of lower mental health status within a decade of living in Canada [18-22] In addition, there is also evidence for an “age at immigration effect” for mental health, where those who immigrated before the age of 18 years old have a higher risk of depression [13].

The majority of immigrants arrive in Canada during their working adult life between the ages of 25–64 years old [23]. Age is also an important determinant of mental health in addition to migration, with the risk of mental health issues varying over the life course [24]. While there are published reports on the prevalence rates and factors associated with mental health of South Asian older adults (aged 55 and older) [14], to our knowledge we have no information on other age groupings.

To date, there are no prevalence statistics for mental health outcomes of South Asian populations across Canada (other than older adult populations). Moreover, most of the research conducted so far on South Asian and immigrant populations has focused solely on the mental health outcomes of depression or self-reported mental health status, neglecting anxiety disorders (the most common mental illness) and self-reported life stress (a particularly salient self-reported mental health measure for immigrants considering the stressors of migration and resettlement and potentially less stigmatized). It is important to examine the risk factors and protective factors of mental health outcomes for South Asian populations through quantitative epidemiological analysis, to add onto research findings to date that have been mostly qualitative in nature. In addition, South Asian populations self-identified the generational culture clash between parents and youth and migration as significant mental health stressors [10]; however, no research in Canada has compared the prevalence and determinants of mental health for first-generation immigrant and second-generation Canadian-born South Asian populations.

Thus, the objective of this study is to determine the estimated prevalence rates, risk factors, and protective factors of mental health outcomes (mood disorders, anxiety disorders, self-perceived mental health, and self-perceived life stress) among the adult South Asian first-generation immigrant and second-generation Canadian-born populations aged 25–64 years old in Canada and assess if the prevalence and determinants are different for these two migration generations.

## Methods

### Data source - Canadian Community Health Survey (CCHS)

Statistics Canada conducts the annual Canadian Community Health Survey (CCHS) to collect health-related data from January to December of each year, surveying individuals over the age of 12 in all provinces and territories, excluding those residing on Indian Reserves, institutions, remote regions, and full-time members of the Canadian Forces. In order to administer surveys to a variety of households, the CCHS samples households by area framing, telephone list framing, and random digit dialing. Response to the survey is voluntary with a response rate of about 69% [25].

### Sample population

This study examined data on South Asian populations across five CCHS cycles from 2007–2011 (unweighted n = 3918; weighted n = 5,962,903). Those between the ages of 25–64 years old (the working adult population) were selected for analysis as this age group comprises of the majority of the immigrant population in Canada. Individuals who responded yes to either of the two following questions were selected as South Asian: “To which ethnic or cultural groups did your ancestors belong? South Asian?” and “You may belong to one or more racial or cultural groups on the following list: South Asian?” The South Asian sample was stratified by immigrant status. South Asian immigrants were selected based on the derived variable Immigration Flag: yes, immigrant; not an immigrant, while the Canadian-born South Asian sample was selected using the survey question: In what country were you born? Canada. (yes, no).

### Outcome variables

Four different mental health outcomes were analyzed (both self-reported clinically diagnosed and self-perceived variables). The presence of mood disorders (depression, bipolar disorder, mania or dysthymia) and anxiety disorders (phobia, obsessive- compulsive disorder or a panic disorder) were assessed based on questions where participants were asked if they had such disorders diagnosed by a health professional (yes, no). Two self-perceived measures of mental health were also analyzed. Self-perceived mental health was examined using the question, “In general, would you say your mental health is excellent, very good, good, fair, or poor?” The outcome was recategorized into a dichotomous variable: self-perceived mental health fair-poor (yes (fair-poor), no (good-excellent)) [19,21]. Lastly, self-perceived life stress was assessed using the survey question, “Thinking about the amount of stress in your life, would you say that most days are: not at all stressful, not very stressful, a bit stressful, quite a bit stressful, or extremely stressful?” This variable was also recategorized into a dichotomous outcome: self-perceived life stress extremely stressful (yes (quite a bit-extremely), no (not at all-a bit)) [26].

### Covariate variables

The following sociodemographic variables were analyzed as potential determinants of mental health outcomes: gender (male, female) and age (25–44, 45–64 years old). The following social support variables were analyzed: marital status (married/common-law, not married), sense of belonging to community (very strong-somewhat strong, somewhat weak-very weak), and the number of children under the age of 12 within the household (none, 1 or more). Indicators of socioeconomic status (SES) were also included: highest level of education (highschool graduate or less, some post-secondary or more), income adequacy (low income household, not low income household) [27,28], food security (food secure, food insecure status), and working status last week (employed, not employed/unable to find job/permanent unable). Lastly, health and behavioral factors were also examined: self-rated health (good-excellent, poor-fair), chronic conditions (having cardiac disease, hypertension, diabetes, chronic obstructive pulmonary disorder, or arthritis) (none, 1 or more), having a regular medical doctor (yes, no), physical activity (active, inactive), and current smoking status (yes, no). For the South Asian immigrant sample, three additional variables of acculturation were examined: years since immigration (0–9 years, 10+ years), age at time of immigration (≤17 years, 18+ years), and language of CCHS interview (English or French, non-official language).

### Data analysis

The estimated prevalence rates (percentages) of the four mental health outcomes were calculated for South Asian immigrant and South Asian Canadian-born populations using the CCHS 2011. Merged data files create an artificial population, which is not the best for calculating descriptive statistics [29] and therefore the unmerged and most recent CCHS data was used (CCHS 2011). CCHS sample weights were applied, estimated prevalence rates were bootstrapped, and 95% confidence intervals (95% CI’s) were reported. CCHS weights are calculated based on how many people each person surveyed represents in the population [30]. Following a similar method to the one used to determine the CCHS sampling design, these weights are then post-stratified according to demographic data [30]. Sample weights are applied to survey data in order to make inferences to the Canadian population [29]. Since the CCHS follows a complicated multi-stage survey design, the re-sampling method involved in bootstrapping ensures that the best calculation of the variance between estimates is made [27]. Sample weights are re-calculated for simple random samples taken repeatedly from the CCHS dataset repeatedly [30]. These weights are then post-stratified for each stratum [30]. This process is repeated 500 times and results in the calculation of 500 bootstrapped weights for each cycle of the CCHS [30]. Statistics Canada has developed programming to carry out these calculations. The application of bootstrapped weights to the inferential statistics was done in consultation with analysts at Statistics Canada.

To determine the factors associated with the mental health outcome measures, data was merged across five separate CCHS cycles 2007–2011 (merged data can be used for modeling). Four multivariate logistic regression models were run separately for the South Asian immigrant population and South Asian Canadian-born population examining the four dichotomous mental health outcomes. Significance was set at p < 0.05. Odds ratios (OR’s) and 95% confidence intervals (CI’s) were reported. CCHS sample weights were applied and the results of the regression modeling were bootstrapped. Preliminary data analysis was carried out using IBM SPSS version 21, while the application of sample weights and bootstrapping of results from the descriptive and multivariate regression analysis were conducted using STATA version 12. Data analysis was conducted at the Statistics Canada York University Research Data Centre and approved for release.

## Results

### Sample characteristics

The sample characteristics of South Asian Canadian-born (unweighted n = 97; weighted n = 265,056) and immigrant populations (unweighted n = 682; weighted n = 997,706) in the CCHS 2011 are displayed in Table 1. In the CCHS 2011, 2.18% of the Canadian population surveyed between the ages of 25–64 years old belonged to South Asian populations. Analysis of sample demographics revealed that in 2011, the South Asian Canadian-born population was significantly younger, less likely to be married/common-law, more likely to have no children, less educated, less likely to experience food insecurity, less likely to have chronic conditions, and more physically active compared to their South Asian immigrant counterparts. Many of these differences may be due to the age difference between the South Asian Canadian-born and immigrant populations.

**Table 1:** Sample characteristics of South Asian Canadian-born and South Asian immigrant populations in Canada aged 25–64 years old, CCHS 2011

		**South Asian Canadian-born weighted n (%) 265,056 (21.0%)**	**South Asian Immigrant weighted n (%) 997,706 (79.0%)**	**p-value**
SOCIODEMOGRAPHIC				
Gender	Male	147,173 (55.5)	530,978 (53.2)	0.480
	Female	117,884 (44.5)	466,728 (46.8)	
Age	25-40 years old	96,597 (89.7)	530,978 (53.2)	0.0001
	41-64 years old	11,090 (10.3)	466,728 (46.8)	
SOCIAL SUPPORT				
Marital status	Married/common-law	75,850 (28.9)	774,264 (77.7)	0.0001
	Not married	186,888 (71.1)	222,858 (22.4)	
Sense of belonging to community	Strong	197,034 (76.0)	720,613 (74.8)	0.693
	Weak	62,332 (24.0)	242,235 (25.2)	
Number of children in household under age of 12	None	190,258 (71.8)	610,579 (61.2)	0.0001
	1 or more	74,799 (28.2)	387,127 (38.8)	
SES				
Highest level of education	HS grad or less	96,109 (37.2)	267,390 (27.3)	0.002
	Some post-sec or more	162,056 (62.8)	711,560 (72.7)	
Income adequacy	Low income household	119,744 (45.2)	332,091 (33.3)	0.0001
	Income adequate	145,313 (54.8)	665,615 (66.7)	
Food security	Food secure	247,441 (96.3)	896,756 (92.6)	0.008
	Food insecure	9,569 (3.7)	72,021 (7.4)	
Working status last week	Employed	160,215 (69.0)	580,261 (61.6)	0.027
	Not employed	72,117 (31.0)	362,495 (38.5)	
HEALTH & BEHAVIOR				
Self-rated health status	Poor-fair	235,978 (89.2)	905,151 (91.0)	0.354
	Good-excellent	28,680 (10.8)	89,311 (9.0)	
Chronic conditions	No conditions	248,349 (93.7)	733,716 (73.5)	0.0001
	1 or more conditions	10,708 (6.3)	263,990 (26.5)	
Regular medical doctor	Yes	228,199 (86.1)	835,919 (83.9)	0.351
	No	36,858 (13.9)	159,975 (16.1)	
Physical activity level	Active	167,736 (64.4)	431,643 (44.5)	0.0001
	Inactive	92,793 (35.6)	539,461 (55.6)	
Current smoking status	Yes	20,791 (7.9)	75,326 (10.2)	0.495
	No	242,929 (92.1)	663,559 (89.8)	
ACCULTURATION				
Years since immigration	0-9 years	—	378,199 (37.9)	—
	10+ years	—	619,507 (62.1)	—
Age at time of immigration	≤ 17 years	—	205,764 (20.8)	—
	18+ years	—	783,294 (79.2)	—
Language of CCHS interview	English or French	—	972,073 (97.4)	—
	Not English or French	—	25,633 (2.6)	—

### Estimated prevalence rates of mental health outcomes

In 2011, 3.48% (95% CI 3.41-3.55%) of South Asian Canadian-born and 3.49% (95% CI 3.46-3.53%) of South Asian immigrant populations reported a diagnosed mood disorder (there was no significant difference). However, South Asian Canadian-born and South Asian immigrant samples varied significantly on all other mental health outcome measures. South Asian immigrant populations had a higher estimated prevalence rate of anxiety disorders (3.44%; 95% CI 3.41-3.48%) compared to South Asian Canadian-born samples (1.09%; 85% CI 1.05-1.13%). South Asian immigrant populations also reported significantly higher estimated prevalence rates of extremely stressful life stress (2.63%; 95% CI 2.60-2.66%) than their South Asian immigrant counterparts (2.35%; 95% CI 2.30-2.41%). In contrast, South Asian Canadian-born populations had a higher estimated prevalence rate of fair-poor self-reported mental health status (4.39%; 95% CI 4.31-4.47%) compared to South Asian immigrant populations (3.44%; 95% CI 3.41-3.48%). Estimated prevalence rates of the four mental health outcomes for South Asian Canadian-born and immigrant populations are displayed in Table 2.

**Table 2:** Estimated prevalence of mental health outcomes for South Asian Canadian-born and South Asian immigrant populations in Canada aged 25–64 years old, CCHS 2011

	**Mood disorder % (95% CI)**	**Anxiety disorder % (95% CI)**	**Poor-fair self-perceived mental health % (95% CI)**	**Extremely stressful self-reported life stress % (95% CI)**
South Asian Canadian-born	3.5 (3.4-3.6)	1.1 (1.1-1.1)	4.4 (4.3-4.5)	2.4 (2.3-2.4)
South Asian Immigrant	3.5 (3.5-3.5)	3.4 (3.4-3.5)	3.4 (3.3-3.4)	2.6 (2.6-2.7)
p-value	0.771	0.0001*	0.0001*	0.0001*

*Indicates significant difference in estimated prevalence rates between South Asian Canadian-born and South Asian immigrant sample.

### Characteristics of mental health outcomes

#### Multivariate logistic regression modeling

In order to increase power, data from CCHS cycles 2007–2011 were merged for multivariate logistic regression analysis. In the CCHS 2007–2011, 2.12% of the Canadian population surveyed between the ages of 25–64 years old belonged to South Asian populations. Multivariate logistic regression modeling revealed different factors associated with mental outcomes for South Asian Canadian-born (unweighted n = 523; weighted n = 1,223,141) and South Asian immigrant populations (unweighted n = 3395; weighted n = 4,739,762) (Table 3). Due to small sample size, food security status and self-rated health status were omitted from the South Asian Canadian-born models.

**Table 3:** Characteristics of mental health outcomes of mental health outcomes for the adult (25–64 years old) South Asian Canadian-born and South Asian immigrant populations, CCHS 2007-2011

	**Presence of a mood disorder**		**Presence of an anxiety disorder**	
	**Canadian-born OR (95% CI)**	**Immigrant OR (95% CI)**	**Canadian-born OR (95% CI)**	**Immigrant OR (95% CI)**
SOCIO DEMO GRAPHIC				
Gender	0.7 (0.2-2.1)	**2.8 (1.5-5.1)**	1.9 (0.6-5.8)	1.4 (0.7-2.9)
Male (ref)				
Female				
Age	1.1 (0.3-4.6)	1.7 (0.9-3.1)	1.1 (0.2-7.3)	1.4 (0.7-2.9)
25-40				
41-64 (ref)				
SOCIAL SUPPORT				
Marital status	1.0 (0.2-4.4)	0.5 (0.2-1.2)	1.9 (0.4-10.2)	0.8 (0.3-2.3)
Married/common-law (ref)				
Not married				
Sense of belonging to community	0.9 (0.3-2.5)	1.1 (0.6-2.0)	1.2 (0.4-4.0)	1.0 (0.5-2.1)
Strong (ref)				
Weak				
Number of children in household under age of 12	1.5 (0.5-5.0)	0.6 (0.3-1.1)	1.4 (0.3-6.0)	1.0 (0.4-2.6)
None (ref)				
1 or more				
SOCIOECONOMIC STATUS				
Highest level of education	0.4 (0.09-2.1)	0.8 (0.5-1.5)	1.8 (0.5-5.9)	0.6 (0.3-1.5)
≤ HS grad				
≥ some post-sec (ref)				
Income adequacy	3.6 (0.8-15.5)	1.4 (0.8-2.3)	2.6 (0.7-10.0)	1.3 (0.6-2.6)
Low income household				
Income adequate (ref)				
Food security	#	**2.7(1.5-5.0)**	#	1.8(0.7-4.5)
Food insecure				
Food secure (ref)				
Working status last week	**2.8 (1.0-7.6)**	1.3 (0.7-2.3)	1.5 (0.6-4.3)	1.9 (0.9-3.9)
Employed (ref)				
Not employed				
HEALTH & BEHAVIOR				
Self-rated health status	#	**4.5 (2.4-8.4)**	#	**5.3 (2.7-10.4)**
Good-excellent (ref)				
Poor-Fair				
Presence of chronic conditions	1.9 (0.6-6.3)	1.7 (0.9-3.1)	1.8 (0.4-8.9)	1.4 (0.7-2.9)
No conditions (ref)				
1 or more conditions				
Having a regular medical doctor	2.1 (0.4-9.7)	0.4 (0.09-1.5)	**0.2 (0.04-0.7)**	0.5 (0.1-1.5)
Yes (ref)				
No				
Physical activity level	**3.6 (1.5-8.7)**	1.0 (0.6-1.8)	2.7 (0.9-7.9)	1.4 (0.7-2.8)
Active (ref)				
Inactive				
Current smoking status	1.2 (0.4-3.6)	**2.6 (1.2-5.6)**	3.4 (0.9-12.4)	2.2 (1.0-5.2)
Yes				
No (ref)				
ACCULTURATION				
Length of stay in Canada	—	0.8 (0.4-1.4)	—	1.0 (0.4-2.4)
0-9 years (ref)				
10+ years				
Age at time of immigration	—	**2.6 (1.2-5.3)**	—	**3.0 (1.4-6.5)**
≤ 17 years old				
18+ years old (ref)				
Language of CCHS interview	—	0.6 (0.1-2.2)	—	0.6 (0.1-3.5)
English or French (ref)				
Not English or French				
	**Poor-fair self- perceived mental health status**		**Extremely stressful self-reported life stress**	
	**Canadian-born OR (95% CI)**	**Immigrant OR (95% CI)**	**Canadian-born OR (95% CI)**	**Immigrant OR (95% CI)**
SOCIODEMOGRAPHIC				
Gender	0.6 (0.2-1.7)	1.1 (0.6-1.9)	1.3 (0.31-5.9)	0.6 (0.4-1.1)
Male (ref)				
Female				
Age				
25-40				
41-64 (ref)	1.3 (0.3-5.2)	1.4 (0.7-2.8)	1.5 (0.4-6.8)	0.7 (0.3-1.2)
SOCIAL SUPPORT				
Marital status	0.8 (0.2-3.4)	0.5 (0.2-1.3)	0.3 (0.04-1.7)	0.6 (0.2-1.5)
Married/common-law (ref)				
Not married				
Sense of belonging to community	1.1 (0.4-3.0)	1.6 (0.9-2.6)	1.7 (0.3-8.6)	1.0 (0.6-1.8)
Strong (ref)				
Weak				
Number of children in household under age of 12	2.1 (0.6-7.1)	**0.5 (0.3-0.9)**	0.8 (0.2-2.8)	1.1 (0.6-2.0)
None (ref)				
1 or more				
SOCIOECONOMIC STATUS				
Highest level of education	0.3 (0.07-1.6)	1.0 (0.6-1.7)	2.3 (0.6-8.6)	1.4 (0.8-2.5)
≤ HS grad				
≥ some post-sec (ref)				
Income adequacy	2.6 (0.5-12.7)	1.2 (0.7-2.1)	1.5 (0.5-3.9)	0.7 (0.4-1.6)
Low income household				
Income adequate (ref)				
Food security	#	**4.3 (2.4-7.9)**	#	**5.8 (2.7-12.7)**
Food insecure				
Food secure (ref)				
Working status last week	**3.2 (1.1-9.3)**	1.1 (0.7-1.9)	1.5 (0.5-5.0)	0.9 (0.5-1.8)
Employed (ref)				
Not employed				
HEALTH & BEHAVIOR				
Self-rated health status	#	**8.7 (5.0-15.4)**	#	**2.9 (1.5-5.7)**
Good-excellent (ref)				
Poor-Fair				
Presence of chronic conditions				
No conditions (ref)	1.8 (0.6-5.5)	2.0 (1.0-3.8)	0.6 (0.2-2.2)	**2.2 (1.3-3.7)**
1 or more conditions				
Having a regular medical doctor	2.3 (0.6-9.5)	0.5 (0.2-1.5)	**0.2 (0.03-0.9)**	1.2 (0.6-2.3)
Yes (ref)				
No				
Physical activity level	**3.2 (1.3-8.1)**	1.3 (0.7-2.3)	1.6 (0.5-5.2)	1.3 (0.7-2.4)
Active (ref)				
Inactive				
Current smoking status	0.6 (0.2-1.7)	**3.0 (1.6-5.9)**	0.9 (0.2-3.6)	1.8 (0.8-4.1)
Yes				
No (ref)				
ACCULTURATION				
Length of stay in Canada	—	1.0 (0.6-1.8)	—	1.2 (0.7-2.1)
0-9 years (ref)				
10+ years				
Age at time of immigration	—	1.9 (0.9-3.8)	—	**3.6 (1.9-6.6)**
≤ 17 years old				
18+ years old (ref)				
Language of CCHS interview	—	1.8 (0.5-6.0)	—	**0.2 (0.03-1.7)**
English or French (ref)				
Not English or French				

#Omitted from model due to low sample size.

Boldface indicates significant difference between Canadian-born and immigrant groups (p < 0.05, 95% CI does not cross 1).

#### Mood disorders

For Canadian-born South Asians, those who were not employed (OR 2.8; 95% CI 1.0-7.6) and those who were physical inactive (OR 3.6; 95% CI 1.5-8.7) were at a significantly increased odds of reporting a diagnosed mood disorder. In contrast, for immigrant South Asians, females (OR 2.8; 95% CI 1.5-5.1), those experiencing food insecurity (OR 2.7; 95% CI 1.5-5.0), those with self-rated fair-poor health status (OR 4.5, 95% CI 2.4-8.4), current smokers (OR 2.6; 95% CI 1.2-5.6), and those who immigrated at the age of 17 or younger (OR 2.6; 95% CI 1.2-5.3) were at a significantly higher risk of mood disorders.

#### Anxiety disorders

Not having a regular medical doctor (OR 0.2; 95% CI 0.04-0.7) was the only significant factor associated with decreased odds of anxiety disorders for Canadian-born South Asian populations. While fair-poor self-rated health status (OR 5.3; 95% CI 2.7-10.4) and immigrating at the age of 17 or younger (OR 3.0; 95% CI 1.4-6.5) were the significant factors associated with increased odds of anxiety disorders for South Asian immigrant populations.

#### Poor-fair self-perceived mental health status

For South Asian Canadian-born populations, not being employed (OR 3.2; 95% CI 1.1-9.3) and being physically inactive (OR 3.2; 95% CI 1.3-8.1) were significantly associated with a greater risk of reporting fair-poor mental health status. On the other hand, for South Asian immigrant populations, having one or more children in the household under the age of 12 (OR 0.8, 95% CI 0.2-2.9) was significantly associated with a decreased odds of reporting fair-poor mental health status, while experiencing food insecurity (OR 4.3; 95% CI 2.4-7.9), poor-fair self-rated health status (OR 8.7; 95% CI 5.0-15.4), and being a current smoker (OR 3.0; 95% CI 1.6-5.9) were significant factors associated with a higher odds of self-reporting poor-fair mental health status.

#### Extremely stressful self-reported life stress

Similar to anxiety disorders, not having a regular medical doctor was the only significant factor associated with a decreased odds reporting extremely stressful life stress for Canadian-born South Asian populations. In contrast, experiencing food insecurity (OR 5.8; 95% CI 2.7-12.7), self-rating health status as poor-fair (OR 2.9; 95% CI 1.5-5.7), reporting one or more chronic conditions (OR 2.2; 95% CI 1.3-3.7) and immigrating at the age of 17 or younger (OR 3.6; 95% CI 1.9-6.6) were significantly associated with higher odds of self-reporting extremely stressful life stress levels for South Asian immigrant populations. Moreover, carrying out the CCHS survey in a non-official language was significantly associated with a decreased odds (OR 0.2; 95% CI 0.03-1.7) of reporting extremely stressful life stress for South Asian immigrant populations.

## Discussion

This study found a varying pattern of mental health for South Asian immigrant population compared to Canadian-born populations. South Asian immigrants experience higher estimated prevalence rates of diagnosed anxiety disorders and self-reported extremely stressful life stress. Moreover, South Asian Canadian-born and South Asian immigrant populations do not vary significantly in estimated prevalence rates of mood disorders. Lastly, South Asian Canadian-born populations have a higher estimated prevalence rate of poor-fair self-perceived mental health status compared to their immigrant counterparts.

Socioeconomic status, acculturative, and health and behavioral factors emerged as important social determinants of mental health outcomes in South Asian populations. Working status, physical activity level, and having a regular medical doctor were the three recurring factors associated with mental health outcomes for South Asian Canadian-born populations. In contrast, South Asian immigrant populations had much greater variety in the risk factors and protective factors for mental health outcomes. Food security status, self-rated health status, and age at time of immigration were the recurring factors associated with mental health outcomes for South Asian immigrant populations.

This study examined the mental health of South Asian populations in Canada at the intersection of immigrant status and found important differences in the estimated prevalence rates and determinants of mental health outcomes. The findings stress the importance of not painting the mental health of all South Asian populations with the same brush. Caution needs to be exercised in treating South Asian populations in Canada as a monolithic entity; rather we need to view them as multiple populations with unique mental health needs. The heterogeneity of South Asian populations cannot be underemphasized. While this study focused on South Asian ethnicity at the intersection of immigrant status, South Asian populations differ along many other axes such as religion, country of origin, and language. Future studies need to examine South Asian mental health in specific sub-populations (e.g. Tamil-speaking Sri Lankan Hindu populations). An intersectional approach to research [31,32] can help ensure that these important contextual factors are taken into consideration.

The differences in estimated prevalence rates of mental health outcomes for South Asian populations highlight the importance of tailoring mental health outreach to specific South Asian sub-populations in Canada. Our findings suggest that anxiety disorders and life stress may be particular areas of concern for South Asian immigrant populations. The estimated prevalence rates of anxiety disorders and extremely stressful life stress may be related to the stressors of migration and acculturating to a new land [10,17]. Programs to deal with anxiety and stress management may be targeted toward South Asian immigrant populations as they had significantly higher prevalence rates of anxiety disorders and high life stress compared to their Canadian-born counterparts. Taking the findings from the multivariate logistic regression analysis, stress management programs for South Asian immigrants could potentially target those living in food insecurity, those with one or more chronic condition, and those who immigrated to Canada before reaching adulthood. Patterson et al. [33] found the highest prevalence rates and risk of mood disorders, anxiety disorders and substance abuse amongst those who had immigrated to Canada before the age of 6 even after adjusting for age, sex, region of origin, marital status, urbanicity, household income, and household size. Mental health programming needs to concentrate on those who migrate to Canada in early childhood as they are at a greater risk for mental health issues. In Guzder, Yohannes, and Zelkowitz’s [34] study comparing Canadian-born and immigrant parents of children with mental health issues in Montreal, immigrant parents were more likely to report barriers in accessing mental healthcare, including the dearth of family doctors and presence of language barriers. Kirmayer et al.’s [35] concluded that cultural or language barriers may be related to the lower prevalence rates of mental health service utilization for immigrants compared to Canadian-born populations in Montreal. Targeted mental health promotion is needed to address these gaps. This study found important differences in the South Asian immigrant and South Asian Canadian-born population aged 25–64 years old, which may be related to mental health outcomes. South Asian Canadian-born populations had significantly higher proportions of those who were younger (25–44 years old), not married, had no children, less educated (high school education or less), experiencing income inadequacy, food insecurity, and not currently employed. On the other hand, South Asian Canadian-born populations had higher proportions of those who reported no chronic conditions and were more physically active. Many of these factors may be related to the younger age of South Asian Canadian-born populations. However, the SES indicators that emerged may have important implications. Food insecurity was the most significant factor associated with extremely stressful life stress for South Asian immigrants. Increased economic assistance from the government for immigrants may help to alleviate this. Moreover, the SES indicator of working status emerged as an important factors related to mental health for South Asian Canadian-born populations. Further research into the impact of SES on mental health for South Asian sub-populations is warranted.

This study found that there was no gender difference in mood disorder risk for Canadian-born South Asians but females were found to have a greater risk of mood disorder than males amongst South Asian immigrant populations. Golding and Burnam [36] carried out stepwise multivariate regression modeling of depression in US-born and immigrant Mexican populations and found that after controlling for social integration, the gender difference in levels of depressive symptomology disappeared. It may be that for the Canadian-born South Asian population the inclusion of other sociodemographic and health factors in the model led to the disappearance of the association between gender and mood disorders, but this did not occur for the South Asian immigrant sample. Further research into this interesting finding, perhaps with stepwise regression analysis, would be helpful.

Self-perceived fair-poor mental health status was the only mental health outcome in which Canadian-born South Asians fared worse than their immigrant counterparts. Despite having similar rates of mood disorders and lower rates of anxiety disorders and extremely stressful life stress, Canadian-born South Asians still perceived their mental health more negatively than South Asian immigrant populations. Further investigation into additional mental health outcomes and reasons for this negative self-perception need to be carried out.

Different characteristics associated with mental health outcomes also emerged in this study for South Asian immigrant and Canadian-born populations. To ensure the delivery of focused and tailored mental health services, the different protective factors and risk factors for South Asian sub-populations need to be considered. For example, female immigrant South Asians were at almost a three-fold greater risk of mood disorders in comparison to their male counterparts (this sex difference where women are at a greater risk of mood disorders has been established in the literature cross-nationally [37]). Mental healthcare professionals and outreach programs for mood disorders can use this information to target female South Asian immigrant populations knowing that this is a particularly at-risk population. Mood disorder mental health programs tailored to South Asian immigrant populations could target women, those living in food insecurity, current smokers, and those who immigrated before the age of 18. On the other hand, mood disorder programs tailored for South Asian Canadian-born populations may potentially target those who are currently not working and not physically active. Socioeconomic status factors such as food security and working status emerged as important characteristics associated with mental health outcomes for South Asian populations. Poverty, low income, unemployment, and precarious employment are important social determinants of mental health [17,37-40]. These factors seem to not only be a problem for South Asian immigrant populations new to Canada, but Canadian-born populations as well. Poverty alleviation, job placement, job skills-matching, professional mentorship and educational programs need to be bolstered in Canada to promote a more equitable job market [38]. Self-rated health status also emerged as a recurring characteristic associated with mental health outcomes. The literature corroborates the association of this factor with mental health [13,41]. Taking the highly stigmatized nature of mental health into consideration and since health encompasses both physical and mental health, self-rated health may be a question mental health professionals can use with South Asian populations as a starting point as it is a potentially less stigmatized question that may be able to point to deeper mental health issues. Those who did not have a regular medical doctor were more likely not to self-report anxiety disorder and high life stress risk amongst Canadian-born South Asian populations. It is estimated that about 15.3% of Canadians do not have access to a regular medical doctor (Statistics Canada, [42]). Not having a family doctor that can diagnose and identify mental health issues may lead to underreporting of these mental health outcomes. Finally, the acculturation measure of age at time of immigration was found to be an important factor associated with mental health outcomes for South Asian immigrant populations. Corroborating Wu and Schimmele’s [12] findings, immigrating during adulthood (18+ years old) seems to be protective for mental health. It may be that those who immigrate as adults do not have as difficult of a time acculturating to a new land as they already arrive with a fully developed sense of their culture and identity. On the other hand, those who immigrate during childhood and as youth experience the pangs of being “caught between two cultures” and must struggle to negotiate a new identity in a new land [43]. Interestingly, length of stay in Canada was not associated with any of the four mental health outcomes for South Asian immigrant populations in Canada. Further investigation in the form of longitudinal studies that can follow South Asian newcomers upon arrival in Canada over time would help elucidate the effect of length of stay in Canada on mental health for South Asian immigrant populations.

### Limitations

This study relied upon cross-sectional survey data, which is not ideal to study the time-dependent variable of migration and the healthy immigrant effect. Longitudinal studies that follow cohorts of immigrants and Canadian-born individuals over time may help to better elucidate the impact of migration on South Asian mental health. In addition, the CCHS relies on self-reported data which is subject to recall bias and social desirability bias. The prevalence rates of mental health are reported estimates and not true prevalence estimates, since not all households chose to participate in the CCHS survey (78% response rate) and certain populations were excluded from the CCHS (those residing on Indian Reserves, institutions, remote regions, and full-time members of the Canadian Forces). Members of South Asian populations with the four mental health outcomes analyzed in this study may have been more or less likely to choose to participate in the survey. The stigma against mental illness may lead to underreporting and non-disclosure of diagnoses. In addition, those living in low income situations and newcomers and immigrants to Canada also may be less likely to take part in population health surveys. The population weights developed by Statistics Canada are unable to correct for this selection bias. The odds ratio estimates may lead to underrepresentation of the risk of mental health outcomes in South Asian populations because of this selection bias. Moreover, as with all epidemiological surveys, the prevalence rates calculated are only estimates. Caution needs to be exercised in interpreting them. As South Asian populations are a relatively new community in Canada, the sample size of Canadian-born South Asians was low. Data across five CCHS cycles was merged in order to increase sample size and power, however, the Canadian-born South Asian sample (unweighted n = 523) still remained relatively small. Findings related to this group also need to be interpreted with caution. Moreover, the social support scales available in the CCHS were only administered as optional content for select provinces. As a result, we were limited in the social support variables available to us across Canada. We used marital status, number of children in the household under the age of 12, and sense of belonging to the community as social support variables. Perceived discrimination and racism are important post-migratory variables especially for racialized populations [44]. However, these variables were not available in the CCHS and could not be included in the models. Sense of belonging to the community was used as a proxy measure. In addition, Canadian-born South Asians are still a new population in Canada. As a result, sample sizes for the variables of food security and self-rated health status were too small to allow for inclusion in the multivariate regression models. In the absence of a variable of migration generation in the CCHS, the South Asian Canadian-born population served as a proxy measure of “second-generation” since the Canadian-born South Asian population is still new to Canada. Technically, second-generation South Asians would only be those whose parents were first-generation immigrants to Canada.

### Strengths

The CCHS offers a nationally representative database with a large sample size, allowing modeling such as multivariate logistic regression analysis. The large sample size is also well suited for capturing the heterogeneity of South Asian populations in Canada. To our knowledge this is the only study to conduct a within ethnic group comparison between South Asian immigrant and Canadian-born populations in order to understand mental health differences between migration generations. This study also utilizes multiple measures of mental health outcomes, both clinically diagnosed and self-perceived, to paint a better picture of the mental health of South Asian populations in Canada.

## Conclusions

Migration is an important social determinant of mental health for South Asian populations in Canada. Significant differences in estimated prevalence rates and characteristics of mental health outcomes were found for first-generation immigrants and second-generation/Canadian-born South Asian populations. Contrary to the “healthy immigrant effect,” South Asian Canadian-born and immigrant populations have a much more nuanced pattern of mental health. Life stress and anxiety disorders emerged as important mental health issues for South Asian immigrant populations, while poor self-perceived mental health emerged for South Asian Canadian-born populations. Socioeconomic and health and behavioral factors were most commonly associated with negative mental health outcomes for South Asian Canadian-born populations, whereas, acculturative, socioeconomic, and health and behavioral factors were important characteristics associated with mental health of South Asian immigrant populations. Female gender, having no children under the age of 12 in the household, food insecurity, poor-fair self-rated health status, being a current smoker, immigrating to Canada before the age of 18, and taking the CCHS survey in either English or French was associated with greater risk of negative mental health outcomes for South Asian immigrant populations, while not being currently employed, having a regular medical doctor, and inactive physical activity level were associated with greater risk for South Asian Canadian-born populations. In terms of policy implications, the study findings suggest that South Asian immigrant populations require better economic support and assistance upon arrival in Canada to alleviate food insecurity and mitigate negative mental health outcomes. The effects of this lack of economic and workforce integration were also seen in the second-generation Canadian-born South Asian population, where not being employed emerged as a significant risk factor of negative mental health outcomes. Mental health outreach programs need to be cognizant of the different mental health determinants for South Asian immigrant and South Asian Canadian-born populations to better tailor mental health services to be responsive to the unique mental health needs of South Asian populations in Canada.

## Abbreviations

CCHS: Canadian community health survey; CI: Confidence interval; OR: Odds ratio; SPSS: Statistical package for social sciences.

## Competing interests

The authors declare that they have no competing interests.

## Authors’ contributions

FI was involved in developing the study design, data analysis, and writing of the manuscript. NK was involved in editing the manuscript. HT offered guidance for the study design, analysis, and writing of the manuscript. All authors read and approved the final manuscript.

## Pre-publication history

The pre-publication history for this paper can be accessed here:

http://www.biomedcentral.com/1471-244X/14/154/prepub
